# Modification of In Vitro and In Vivo Antioxidant Activity by Consumption of Cooked Chickpea in a Colon Cancer Model

**DOI:** 10.3390/nu12092572

**Published:** 2020-08-25

**Authors:** María S. Cid-Gallegos, Xariss M. Sánchez-Chino, Isela Álvarez-González, Eduardo Madrigal-Bujaidar, Verónica R. Vásquez-Garzón, Rafael Baltiérrez-Hoyos, Saúl Villa-Treviño, Gloria Dávila-Ortíz, Cristian Jiménez-Martínez

**Affiliations:** 1 Departamento de Ingeniería Bioquímica, Escuela Nacional de Ciencias Biológicas, Instituto Politécnico Nacional, Unidad Profesional Adolfo López Mateos, Zacatenco, Av. Wilfrido Massieu Esq. Cda. Miguel Stampa S/N, Alcaldía Gustavo A. Madero, Mexico City 07738, Mexico; cid.stephanie@gmail.com (M.S.C.-G.); gdavilao@yahoo.com (G.D.-O.); 2 Catedra-CONACyT, Departamento de Salud, El Colegio de la Frontera Sur-Villahermosa, Tabasco 86280, Mexico; xsanchez@ecosur.mx; 3 Laboratorio de Genética, Escuela Nacional de Ciencias Biológicas, Instituto Politécnico Nacional, Unidad Profesional Adolfo López Mateos, Zacatenco, Av. Wilfrido Massieu Esq. Cda. Miguel Stampa S/N, Alcaldía Gustavo A. Madero, Mexico City 07738, Mexico; isela.alvarez@gmail.com (I.Á.-G.); eduardo.madrigal@lycos.com (E.M.-B.); 4 Catedra-CONACyT, Facultad de Medicina y Cirugía, Universidad Autónoma Benito Juárez de Oaxaca, Oaxaca de Juárez 68120, Mexico; veronicavasgar@gmail.com (V.R.V.-G.); rbaltierrez@hotmail.com (R.B.-H.); 5 Departamento de Biología Celular, Centro de Investigación y de Estudios Avanzados del Instituto Politécnico Nacional, Mexico City 07360, Mexico; svilla@cell.cinvestav.mx

**Keywords:** legumes, cooked chickpea, antioxidant activity, oxidation markers, colon cancer

## Abstract

Chickpea has been classified as a nutraceutical food due to its phytochemical compounds, showing antioxidant, anti-inflammatory, and anticancer activity. To investigate this, we evaluated the effect of cooking on the nutritional and non-nutritional composition and the in vitro and in vivo antioxidant activity of chickpea seed. The latter was determined by the variation in the concentration of nitric oxide (NO), oxidized carbonyl groups (CO), malondialdehyde (MDA), and the expression of 4-hydroxy-2-nonenal (4-HNE) in the colon of male BALB/c mice fed with a standard diet with 10 and 20% cooked chickpea (CC). We induced colon cancer in mice by administering azoxymethane/dextran sulfate sodium (AOM/DSS); for the evaluation, these were sacrificed 1, 7, and 14 weeks after the induction. Results show that cooking does not significantly modify (*p* < 0.05) nutritional compounds; however, it decreases the concentration of non-nutritional ones and, consequently, in vitro antioxidant activity. The in vivo evaluation showed that animals administered with AOM/DSS presented higher concentrations of NO, CO, MDA, and 4-HNE than those in animals without AOM/DSS administration. However, in the three evaluated times, these markers were significantly reduced (*p* < 0.05) with CC consumption. The best effect on the oxidation markers was with the 20% CC diet, demonstrating the antioxidant potential of CC.

## 1. Introduction

Chickpea seed (*Cicer arietinum* L.) is a legume rich in complex carbohydrates and quality proteins; therefore, it is a good alternative to consuming animal protein [1]. Since ancient times, man has processed legumes for consumption intending to generate tasty and nutritious products. Among these processes, germination, fermentation, and cooking—either by direct heat or with pressure—stand out. As a result of these processes, sensory and nutritional characteristics are improved [2] since they increase the digestibility and bioavailability of macronutrients [3]. Regarding the cooking process, the method used significantly influences the composition of the macronutrients, micronutrients, and bioactive compounds, depending on their solubility and thermolability [4]. For example, this process inactivates or decreases thermolabile non-nutritive compounds such as protease inhibitors, lectins, and phytic acid [5]. It can also decrease the concentration of vitamins and minerals present in food. In the case of chickpea seed, cooking modifies the composition and quality of its proteins, fats, fibers, minerals, B vitamins (B5, B6, and B9), and vitamin E. This process also reduces the concentration of its phytochemical compounds [4,6,7,8]. Phytochemicals are non-nutritional bioactive compounds found in fruits, vegetables, grains, and legumes. They are considered bioactive compounds since they can reduce the risk of chronic diseases [9] and non-nutritional because they decrease the bioavailability of nutrients [6]. The phytochemical compounds found in chickpea seeds are saponins, phytic acid, lectins, protease inhibitors, amylase inhibitors, bioactive peptides, sterols, dietary fiber, resistant starch, oligosaccharides, unsaturated fatty acids, carotenoids, and isoflavones [10,11].

Due to the phytochemical compounds found in chickpea seeds, these have been used in studies focused on the prevention and control of different chronic non-transmissible diseases such as obesity, cardiovascular diseases, diabetes, inflammation, and cancer [11,12,13,14,15,16]. These diseases are directly related to oxidative stress (OS) [11,14,17,18,19]. OS occurs when the body’s antioxidant systems are insufficient to counteract the activity and quantity of reactive oxygen (ROS) or nitrogen (RNS) species, thus generating functional alterations in various biomolecules [20]. Both ROS and RNS in high concentrations can cause cell damage or death by oxidation of proteins, lipids, and nucleic acids [21]. Consequently, they produce mutations at the DNA level and contribute significantly to the formation and progression of cancer [22].

Carcinogenesis is a multi-stage process. It consists of DNA modification or the formation of a mutated cell, followed by uncontrolled selective growth [23]. The ROS and RNS generated during carcinogenesis modify gene expression, regulate signal transduction pathways, and modulate protein function. Likewise, they promote the activation of enzymes such as inducible nitric oxide synthase (iNOS) [14], cyclooxygenase-2 (COX-2), and prostaglandin E2 (PGE2), as well as the nuclear factor kappa B (NF-kB). All these compounds are related to the promotion of tumor angiogenesis, an essential step in the progression and spread of solid tumors [24,25]. Under OS conditions, an unspecific inflammatory response is generated that promotes the suppression of the immune system, favoring tumor growth. This is due to the combined action of hormones, cytokines, and low-molecular weight second messengers that induce the activation of mast cells and leukocytes. The latter massively release ROS and RNS, including O_2_•, OH•, H_2_O_2_, •NO, and HClO at levels above the toxic threshold [26].

One of the most widely used therapies in carcinogenesis is the treatment with antineoplastic drugs such as alkylating agents (cyclophosphamide), antibiotics (bleomycin), antimetabolites (5-fluorouracil), platinum derivatives (cisplatin), and camptothecin derivatives [27]. Additionally, there are plant-based therapies with purified or synthesized antioxidant compounds such as quercetin, resveratrol, and vitamin E [28]. These antioxidants are found in some foods of plant origin like fruits, vegetables, cereals, and legumes [29,30].

In the case of legumes, Murillo et al. [17] reported that the consumption of chickpea flour decreased by 64% preneoplastic lesions induced with azoxymethane (AOM) in CF-1 mice. They related this result to the activity of Bowman–Birk inhibitors, saponins, and phytosterols found in chickpea seeds. Sánchez-Chino et al. [13] studied the effect of the consumption of cooked chickpea added in 2 and 10% to the diet of ICR mice, in which colon cancer was generated with AOM + dextran sulfate sodium (DSS). They reported that the consumption of cooked chickpea reduced preneoplastic and neoplastic lesions, as well as cell proliferation markers (cell proliferation nuclear antigen (PCNA) and Ki-67). Faris et al. [31] reported that lentil consumption significantly increased glutathione S-transferase activity and inhibited cytochrome P450 activity. The latter is responsible for the metabolism of AOM, which promotes colon carcinogenesis through base alkylation and is highly oxidizing. Zhang et al. [32] evaluated the effect of a diet that included 20% beans in a model of DSS-induced colitis. This diet showed an anti-inflammatory effect correlated with the bean’s fermentable compounds, such as resistant starch, oligosaccharides, non-starch polysaccharides, and phenolic compounds. These authors also reported a reduction in the expression of messenger ribonucleic acid (mRNA) and pro-inflammatory cytokines interleukin 6 (IL-6) and interferon gamma (IFN-γ). Likewise, they reported the overexpression of the anti-inflammatory cytokine interleukin 10 (IL-10) and apoptosis-mediating genes. They also observed an increase in the number of cells in the studied colon tissue and a higher content of short-chain fatty acids, such as butyrate. Moreover, they reported improved integrity of the intestinal barrier, which exerts direct effects on the colon epithelium by modulating signaling pathways related to inflammation and inhibition of histone deacetylase activity. Additionally, some reports have indicated that the consumption of legumes in humans reduces plasma concentrations of inflammatory markers (which generate an oxidizing environment) in overweight, diabetic patients [33,34]. Therefore, this work aimed to evaluate the effect of cooking on the nutritional and non-nutritional composition and the in vitro antioxidant activity of chickpea seed and to evaluate the in vivo antioxidant activity that the consumption of CC exerts in colon carcinogenesis in experimental animals.

## 2. Materials and Methods

### 2.1. Chickpea (Cicer arietinum L.) Seed

Raw chickpea (RC) seeds were purchased from the Central de Abastos of Mexico City. The RC seeds were manually conditioned; for this, foreign matter and seeds in poor condition were separated and eliminated [35]. Subsequently, the selected RC seeds were ground and sieved to obtain a fine flour (50 mesh, 0.297 mm opening). CC was obtained according to the methodology described by Margier et al. [4] and Sánchez-Chino et al. [13] with modifications. First, the RC seeds were soaked in water in a 1:4 ratio (seeds/water) for 12 h. Then, the soaking water was removed, and the RC seeds were placed in a pressure cooker, adding water in a 1:5 ratio. The RC seeds were cooked at 120 °C for 25 min; later, they were allowed to cool, and the cooking water was removed. The seeds were lyophilized (Labconco, Kansas City, MO, USA) with three cycles of 12/12 h, 0.280 mBar, and −33 °C. Finally, the CC seeds were ground and sieved to obtain a fine flour (Mesh 50, 0.297 mm opening).

### 2.2. Characterization of Nutritional and Non-Nutritional Compounds of Chickpea Seed

#### 2.2.1. Nutritional Composition

The proximate chemical composition of the RC and CC flour was determined with the following AOAC methods [36]: moisture (Method 925.10), ash content (Method 923.03), lipids (Method 920.39), protein (Method 920.87), and total dietary fiber (Methods 985.29, 993.21). Total carbohydrates were estimated by difference to 100% of the total compounds [36].

#### 2.2.2. Non-Nutritional Composition

The determination of the main non-nutritional compounds of the RC and CC flours was performed with the following assays: saponins (Luo et al. [37]), phytic acid (Corzo et al. [38]), and trypsin inhibitors (TI) (Sánchez-Chino et al. [13]).

#### 2.2.3. In Vitro Antioxidant Properties

The in vitro antioxidant properties of the RC and CC flours were evaluated with the determination of total phenolic compounds (Zhang et al. [39]) and antioxidant activity. Antioxidant activity was evaluated with the oxygen radical absorbance capacity (ORAC), hydroxyl radical scavenging activity, and superoxide radical scavenging activity. All the assays were adapted for use in a microplate.

ORAC was determined according to Ou et al. [40], with some modifications. The ORAC assay relies on free radical damage caused to a fluorescent probe (fluorescein) by an oxidizing reagent. The result is a loss of fluorescent intensity over time [41]. The area under the fluorescence decay curve (AUC) (Equation (1)) of the sample and the blank (PBS pH 7.4) was calculated.

(1) $$AUC=1+\;\sum_{i=80}\left(\frac{f_{i}}{f_{0}}\right)$$

where $f_{0}$ and $f_{i}$ are the fluorescence readings at time 0 and time i.

The antioxidant effect was determined by calculating the net area under the curve (Net AUC) (Equation (2)).

(2) $$Net\;AUC={AUC}_{sample}-\;{AUC}_{blank}$$

This value was interpolated in a Trolox calibration curve and the result was expressed as mg Trolox eq./g dry base.

Hydroxyl radical (OH^−^) scavenging activity was estimated according to Avellar et al. [42], by generating hydroxyl radicals formed from an oxidation reaction with dimethyl sulphoxide (DMSO) [43]. Results were expressed as percentage of inhibition, which was determined by comparing the sample with a standard (gallic acid).

Superoxide radical scavenging activity (O_2_) was determined as the percentage of inhibition of pyrogallol autoxidation. This was calculated through optical density in the presence or absence of pyrogallol and the sample [44].

### 2.3. In Vivo Antioxidant Activity

#### 2.3.1. Ethical Approval

The ethics committee of Escuela Nacional de Ciencias Biológicas/Instituto Politécnico Nacional (ENCB/IPN) approved the experimental protocol carried out in this research (Approval No. CEI-ENCB-011-2017) on 14 June 2017.

#### 2.3.2. Animals

The animals used for the experimental protocol were BALB/c male mice (*Mus musculus*) (Bioterio of the Universidad Autónoma del Estado de Hidalgo, Hidalgo, Mexico) with the following characteristics: 6–8 weeks of age and weight in the range of 20–25 g. The conditioning period for all mice was seven days with 12 h light/darkness cycles at 23 °C, free of pathogens. During this period, they were supplied with standard laboratory animal feed (Rodent Laboratory Chow 5001, LabDiet, St. Louis, MO, USA) and purified water, both ad libitum.

#### 2.3.3. Cooked Chickpea Diets

CC was used for the in vivo evaluation of antioxidant activity since it is the common form of consumption. CC flour was added to the standard feed for laboratory animals with 10% (Murillo et al. [17]; Sánchez-Chino et al. [13]) and 20% substitution (Monk et al. [45]).

#### 2.3.4. Colon Cancer Induction

Colon cancer induction was performed according to the model proposed by Tanaka [46] with modifications on carcinogenic doses, using AOM (A5486, Sigma-Aldrich, St. Louis, MO, USA) and DSS (36,000–50,000 M.W., MP Grade, CAS 9011-18-1, MP Biomedicals, Montreal, QC, Canada). The process started by applying two intraperitoneal injections of AOM at a concentration of 10 mg/kg body weight, in injectable saline solution, one every five days. The induction continued by administering two cycles of 1.5% *w*/*v* DSS to the mice in the drinking water. Each cycle lasted five days, with three days of rest between each cycle.

#### 2.3.5. Experimental Protocol

**Figure 1:**
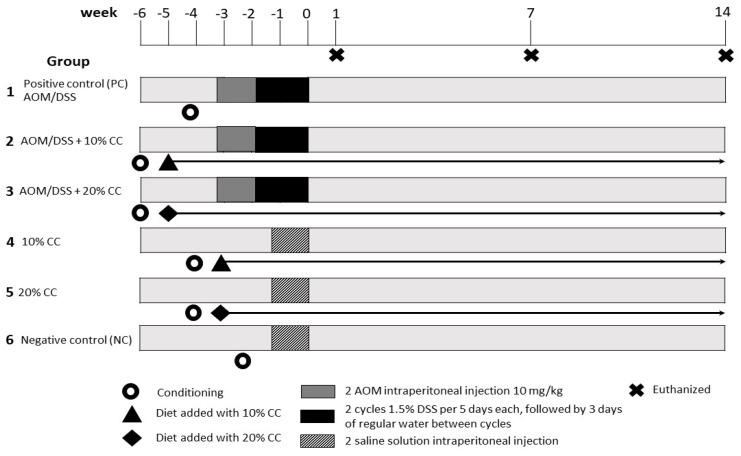
Experimental design of AOM/DSS colon carcinogenesis model to evaluate cooked chickpea (CC) antioxidant activity. NC: negative control; PC: positive control; AOM: azoxymethane; DSS: dextran sulfate sodium; CC: cooked chickpea.

Figure 1 shows the experimental design [46] for the evaluation of the protective effect of CC on the oxidation generated in colon cancer. The design consisted of 6 groups of 21 mice each, randomly selected. Groups 1, 2, and 3 were submitted to the colon cancer induction process previously described. Group 1 maintained a standard food diet until sacrifice, so it served as a positive control (PC) to observe the development of cancer and the oxidation generated. After the conditioning week, Group 1 began the induction of colon cancer. After the conditioning week, Groups 2 and 3 began a CC diet, which they maintained until their sacrifice. Group 2 had a diet with 10% CC in their food [13,17], while Group 3 had 20% in their food [45]. The induction of colon cancer in these groups began two weeks after the CC feeding started. Colon cancer was not induced in Groups 4, 5, and 6. Group 4 had a diet with 10% CC in their food, while Group 5 had 20%. Therefore, these groups were used to determine the possible changes caused by the consumption of CC in the tested doses. Two weeks after CC feeding started, Groups 4 and 5 received two intraperitoneal injections of saline solution, one every five days. Group 6, called negative control (NC), had an exclusive standard food diet; therefore, in this group the basal levels of the markers used for the evaluation of in vivo antioxidant activity are found. After the conditioning period, Group 6 received two intraperitoneal injections of saline solution, one every five days. All groups were euthanized by cervical dislocation. The euthanasia of Groups 1, 2, and 3 began on Weeks 1, 7, and 14 after the completion of DSS administration. The sacrifice of Groups 4, 5, and 6 started on Weeks 1, 7, and 14 after the completion of the saline solution injections application. Subsequently, the colon was removed from each mouse and washed with phosphate-buffered saline (PBS) (pH 7.4) at 4 °C. From each group, the colon of four animals was used for the determination of protein, lipid, and nitric oxide oxidation in homogenized tissue and three animals for the analysis of 4-hydroxy-2-nonenal (4-HNE) by immunohistochemistry. These parameters were used for the evaluation of the protective effect of CC on the oxidation generated in colon cancer.

#### 2.3.6. Determination of Nitric Oxide

Griess reagent was prepared by mixing two solutions, A and B, in a 1:1 ratio. Solution A was obtained by mixing 0.132 g of sulfanilamide with 6 mL of glacial acetic acid in 10 mL of water. For Solution B, 0.01 g of N-(1-Naphthyl)ethylenediamine was dissolved in 10 mL of distilled water. Then, the sample was prepared by weighing 0.2 g of the colon and homogenizing in a 1:4 ratio with PBS (pH 7.4) at 4 °C in an Ultra-Turrax homogenizer (Daigger, T-25, Hamilton, NJ, USA) for 30 s at 5000 rpm. For the determination of nitric oxide (NO), 100 µL of the homogenized colon was mixed with 300 µL of the Griess reagent and 600 µL of distilled water. This mixture was homogenized for 30 s in a vortex, and the absorbance was measured at 540 nm in a spectrophotometer (Thermo Spectronic, 20 Genesys, Rochester, NY, USA). NO concentration was expressed as µmoles of NO/g of tissue [47].

#### 2.3.7. Determination of Oxidized Proteins

For sample preparation, 0.35–0.4 g of the colon was weighed and homogenized in a 1:10 ratio with PBS (pH 7.4) at 4 °C in an Ultra-Turrax homogenizer for 30 s at 5000 rpm. For the determination, 200 µL of the homogenized colon was mixed with 500 µL of DNFH (0.1 g 2,4-Dinitrophenylhydrazine in 2 M HCl to a final volume of 100 mL). The mixture was incubated for 1 h at room temperature in the dark. Subsequently, 500 µL of 20% *v*/*v* trichloroacetic acid (TCA, T6399, Sigma Aldrich, St. Louis, MO, USA) was added and homogenized for 10 s in a vortex. To precipitate the hydrazones generated by the proteins, the mixture was centrifuged at 12,700× *g* for 10 min. The precipitate was washed three times with 1 mL of ethyl acetate-ethanol (1:1). The obtained pellet was re-suspended with 1 mL of 6 M guanidine hydrochloride (G4505, Sigma Aldrich, St. Louis, MO, USA), in phosphate buffer pH 2.3. Then, it was incubated at 37 °C for 15 min and centrifuged at 12,700× *g* for 10 min. The absorbance of the supernatant was measured at 361 nm in a spectrophotometer. The concentration of the oxidized carbonyl groups (CO) was calculated with a molar extinction coefficient of 21,000 M^−1^ cm^−1^ and was expressed as ng/µg of protein of oxidized carbonyls [13].

#### 2.3.8. Determination of Lipid Peroxidation

For sample preparation, 0.35–0.4 g of the colon was weighed and homogenized in a 1:10 ratio with PBS (pH 7.4) at 4 °C in an Ultra-Turrax homogenizer for 30 s at 5000 rpm. For the determination, 500 µL of the homogenized colon was mixed with 2 mL of TCA-TBA-HCl (15 g TCA + 0.3725 g TBA + 2.73 mL HCl in a final volume of 100 mL with distilled water). The mixture was boiled for 15 min, then chilled in an ice bath for 10 min and centrifuged at 2509× *g* for 10 min. The absorbance of the obtained supernatant was measured in a spectrophotometer at 532 nm. Lipid peroxidation was calculated with a molar extinction coefficient of 156,000 M^−1^ cm^−1^ and was expressed as ng of malondialdehyde (MDA) per µg of protein [13].

#### 2.3.9. Immunohistochemistry of 4-HNE

Immunohistochemical analysis of 4-HNE was performed in the medial and distal portions of the colon. These were fixed with 4% formaldehyde for 24 h at 4 °C. Subsequently, the samples were dehydrated in ethanol solutions in increasing concentrations (70, 80, and 92% [13]). Then they were placed in a chloroform/xylol mixture (1:1) for 24 h for embedding in low-melting point paraffin (Paraplast, Leica, Buffalo Grove, IL, USA) at 55 °C [13]. Next, 3 µm cuts were made using a microtome (Leica RM2125 RTS, US). Samples were placed on a slide with 4% 3-aminopropyl-trimethoxysilane in acetone for analysis. The slides were placed in a Coplin with citrate buffer (pH 6), and the antigen retrieval was performed with citrate buffer at 120 °C for 20 min in a pressure cooker. Subsequently, endogenous peroxidase was blocked with H_2_O_2_ in 6% methanol for 30 min and the nonspecific sites with 5% bovine serum albumin (BSA) in PBS (pH 7.4) for 60 min at room temperature. The 4-HNE rabbit polyclonal antibody (ab46545, ABCAM, Cambridge, MA, USA) (diluted 1:50 in PBS [pH 7.4]) was used for immunostaining; then, the samples were incubated overnight at 4 °C. These were washed with PBS (pH 7.4), and then goat anti-rabbit IgG antibody [HRP] (656120, Thermo Fisher, Waltham, MA, USA) diluted (1:200) in 1% BSA in PBS was added. The addition of the chromogen substrate was carried out with 3,3′-Diaminobenzidine (DAB-PLUS substrate kit 00-2020, Life Technologies, Waltham, MA, USA) and counterstaining with Harris’s hematoxylin solution (Cat 738, HYCEL, MX). The tissues were dehydrated at a temperature of approximately 40 °C in a Coplin for 3 min and mounted with resin (Cat 7989, HYCEL, MX,). For analysis, the samples were viewed under a 40X optical microscope. Finally, for all the studied groups, 10 random fields were quantified in the images obtained with the Image J 1.52p software from National Institute of Health, USA.

### 2.4. Statistical Analyses

All results were processed using descriptive statistics like measures of central tendency (mean) ± standard error. With the Minitab 17.0 statistical software, one-way analysis of variance (ANOVA) and Tukey’s comparison test were performed to identify significant differences (*p* ≤ 0.05) between groups.

## 3. Results

### 3.1. Nutritional and Non-Nutritional Composition of RC and CC

Table 1 shows the nutritional and non-nutritional analysis of RC and CC seeds. Carbohydrates and proteins comprise the majority of the components in both cases. After cooking, there was no significant change in these components. However, in ash content and lipids, there were significant changes (*p* < 0.05). Ash content decreased 57%; this effect could be related to the solubility of non-nutritional compounds [3]. By contrast, lipids increased 36% after cooking. This increase may be due to a decrease in the fraction of soluble compounds in the CC, which are transferred to the soaking and cooking water [48]. Corzo et al. [38] reported an increase in lipids in cooked beans, which they attributed to a lipolysis phenomenon that is occasionally catalyzed by processing some foods in the presence of water and high temperature.

Regarding the effect of cooking on the non-nutritional compounds of chickpea, it was observed that this process significantly reduces (*p* < 0.05) its concentration: saponins by 30%, phytates by 19%, and trypsin inhibitors by 85%, due to its chemical properties.

### 3.2. In Vitro Antioxidant Properties of RC and CC

Table 2 shows the in vitro antioxidant properties of RC and CC. The cooking process significantly decreased (*p* < 0.05) the antioxidant activity of chickpea. Evaluated with ORAC, it decreased 48%, with the hydroxyl radical 32%, and with the superoxide radical 39%. Total phenolic compounds were reduced by 25%. This is due to their thermolability, water solubility, and hydrolyzability [49].

### 3.3. In Vivo Antioxidant Activity of CC

#### 3.3.1. Nitric Oxide (NO) Concentration in Colon Homogenates

**Figure 2:**
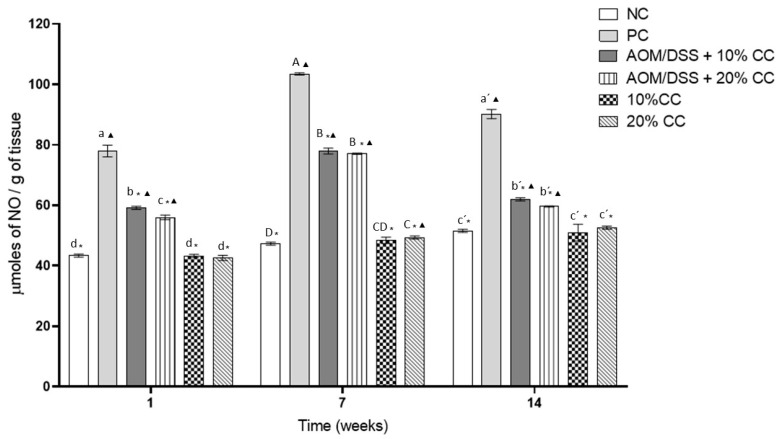
Nitric oxide (NO) content as an indirect measure in colon by effect of oxidation caused by AOM/DSS in BALB/c mice in three periods. Lowercase letters correspond to Week 1, uppercase letters correspond to Week 7, and raw letters correspond to Week 14. * means significant difference from the PC in the same evaluation week and ^▲^ significant difference with respect to the NC in the same evaluation week. One-factor ANOVA (*p* ≤ 0.05), Dunnett’s test. Different letters indicate significant difference (*p* < 0.05) in the same evaluation week by Tukey’s test. Values are presented as the mean ± S.D. from four experimental replicas.

NO is related to inflammation in the tissues, which leads to carcinogenic processes; therefore, the evaluation of NO in the colon was carried out by indirect measurement. Figure 2 shows the NO concentration as a measure of NO in the colon of the analyzed mice. The positive control (PC) had higher concentrations of NO than the negative control (NC) due to the administration of AOM/DSS in the first one.

NO concentrations in the PC were 80, 118, and 75% higher in Weeks 1, 7, and 14 compared to the NC. On the other hand, NO concentrations in the AOM/DSS + 10% CC group were 24, 25, and 31% lower on Weeks 1, 7, and 14 compared to the PC. NO concentrations in the AOM/DSS + 20% CC group were 28, 25, and 34% lower on Weeks 1, 7, and 14 compared to the PC. Finally, the groups not treated with carcinogens, which maintained supplemented diets with 10 and 20% CC, showed similar concentrations to the basal ones, NC, at each studied time.

#### 3.3.2. Quantification Oxidized Carbonyl Groups (CO) from Proteins in Colon Homogenates

**Figure 3:**
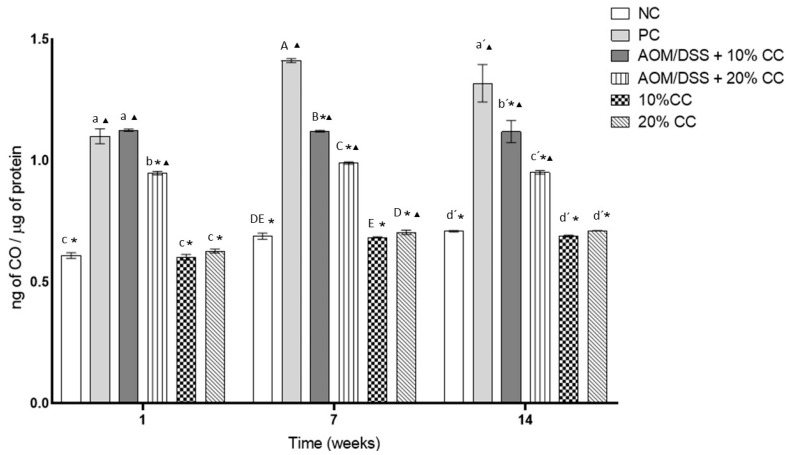
Content of oxidized carbonyl groups (CO) in colon due to the effect of oxidation caused by AOM/DSS in BALB/c mice in three periods. Lowercase letters correspond to Week 1, uppercase letters correspond to Week 7, and raw letters correspond to Week 14. * means significant difference from the PC in the same evaluation week and ^▲^ significant difference with respect to the NC in the same evaluation week. One-factor ANOVA (*p* ≤ 0.05), Dunnett’s test. Different letters indicate significant difference (*p* < 0.05) in the same evaluation week by Tukey’s test. Values are presented as the mean ± S.D. from four experimental replicas.

Figure 3 shows the concentration of CO in the colon of the analyzed mice. This parameter is an indicator of protein oxidation. CO concentrations in the PC were 80, 105, and 86% higher on Weeks 1, 7, and 14 compared to the NC. On the other hand, there was no significant difference in CO concentrations between the AOM/DSS + 10% CC group and the PC on Week 1 of the evaluation. In contrast, in the same evaluation period, CO concentration in the AOM/DSS + 20% CC group was lower by 14% compared to the PC. However, CO concentrations in the AOM/DSS + 10% CC group were lower by 21 and 30% on Weeks 7 and 14 compared to the PC. Similarly, CO concentrations in the AOM/DSS + 20% CC group were lower by 30 and 28% on Weeks 7 and 14 compared to the PC. Finally, CO concentrations in the groups not treated with carcinogens and who maintained supplemented diets with 10 and 20% CC showed concentrations similar to the NC in each analyzed time.

#### 3.3.3. Concentration of MDA from Oxidized Lipids in Colon Homogenates

**Figure 4:**
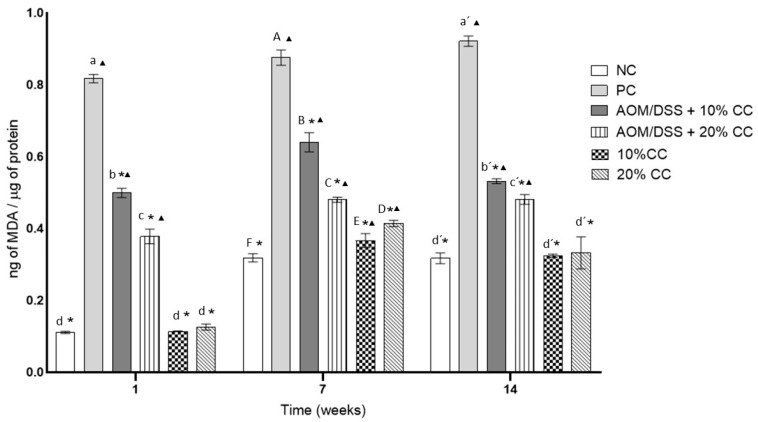
Malondialdehyde (MDA) content in the colon due to the effect of oxidation caused by AOM/DSS in BALB/c mice studied in three periods. Lowercase letters correspond to Week 1, uppercase letters correspond to Week 7, and raw letters correspond to Week 14. * means significant difference from the PC in the same evaluation week and ^▲^ significant difference with respect to the NC in the same evaluation week. One-factor ANOVA (*p* ≤ 0.05), Dunnett’s test. Different letters indicate significant difference (*p* < 0.05) in the same evaluation week by Tukey’s test. Values are presented as the mean ± S.D. from four experimental replicas.

MDA is a product of lipid oxidation, capable of inactivating many cellular proteins by forming cross-links. Figure 4 shows MDA concentrations in the colon of the analyzed mice. In Week 1, the MDA concentration in the PC was seven times higher than in the NC. The concentration of MDA in the PC increased 7 and 12% on Weeks 7 and 14 with respect to the level found on Week 1. On the other hand, on Week 1, compared to the PC, the MDA concentration decreased in the AOM/DSS + 10% CC group by 38% and in the AOM/DSS + 20% CC group by 53%. Then, during Week 7, a beneficial effect on the animals of those groups was observed, since they presented 26% (AOM/DSS + 10% CC) and 45% (AOM/DSS + 20% CC) less MDA concentration. By Week 14, a reduction in MDA was proved in the AOM/DSS + 10% CC group, since it presented 42% less than the PC, while in the case of the AOM/DSS + 20% CC group, there was a 48% reduction compared to the PC. Finally, the MDA concentrations in the groups not treated with carcinogens and who maintained supplemented diets with 10 and 20% CC did not show a significant difference (*p* < 0.05) with respect to the NC at each analyzed time.

#### 3.3.4. Expression of 4-HNE on Colon

**Figure 5:**
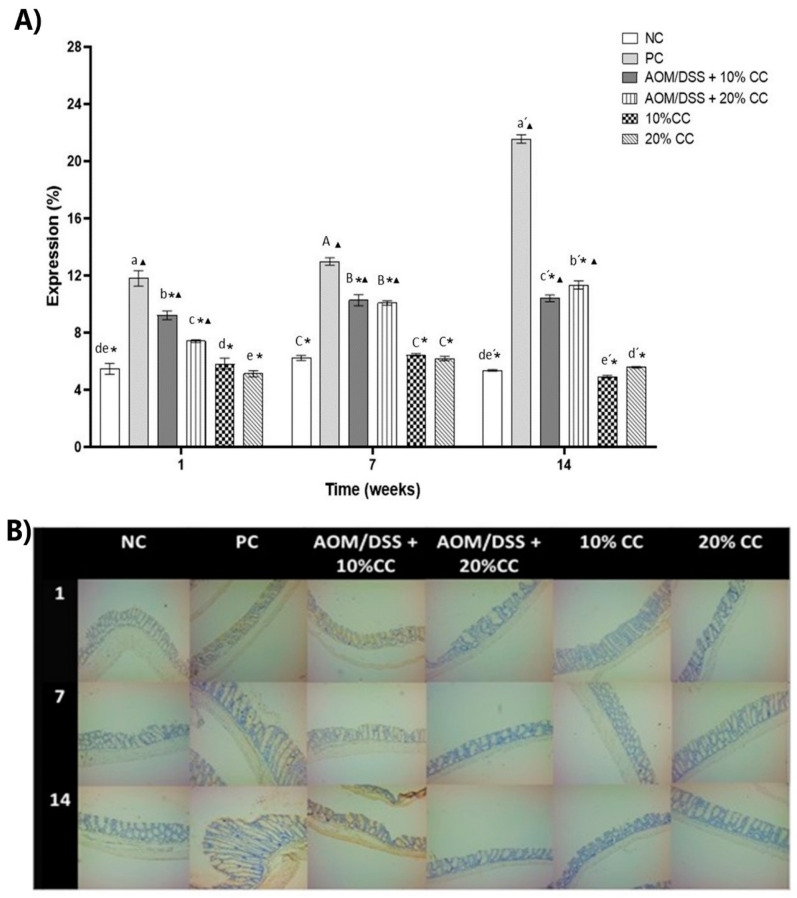
(**A**) Percentage of 4-hydroxy-2-nonenal (4-HNE) expression in the middle portion of the colon of BALB/c mice administered with AOM/DSS in three periods. (**B**) Representative images of the histology of the colonic mucosa by immunohistochemistry in the middle portion on Weeks 1, 7, and 14. Magnification 10×. Lowercase letters correspond to Week 1, uppercase letters correspond to Week 7, and raw letters correspond to Week 14. * means significant difference from the PC in the same evaluation week and ^▲^ significant difference with respect to the NC in the same evaluation week. One-factor ANOVA (*p* < 0.05), Dunnett’s test. Different letters indicate significant difference (*p* < 0.05) in the same evaluation week by Tukey’s test. Values are presented as the means ± S.D. from three experimental replicas.

**Figure 6:**
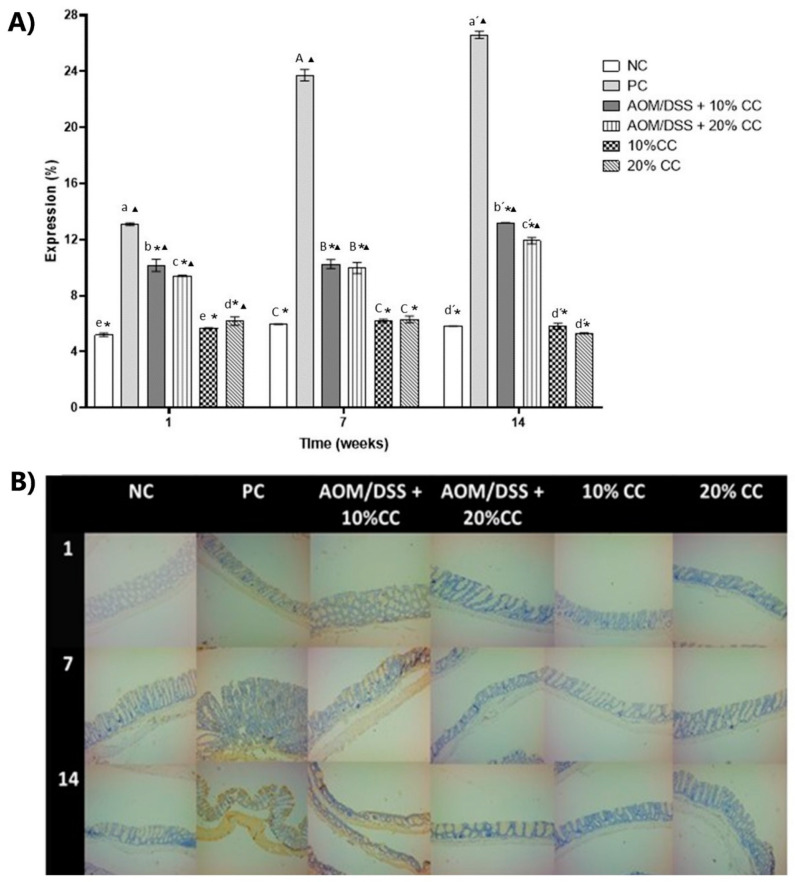
(**A**) Percentage of 4-hydroxy-2-nonenal (4-HNE) expression in the distal portion of the colon of BALB/c mice administered with AOM/DSS in three periods. (**B**) Representative images of the histology of the colonic mucosa by immunohistochemistry in the distal portion on Weeks 1, 7, and 14. Magnification 10×. Lowercase letters correspond to Week 1, uppercase letters correspond to Week 7, and raw letters correspond to Week 14. * means significant difference from the PC in the same evaluation week and ^▲^ significant difference with respect to the NC in the same evaluation week. One-factor ANOVA (*p* < 0.05), Dunnett’s test. Different letters indicate significant difference (*p* < 0.05) in the same evaluation week by Tukey’s test. Values are presented as the means ± S.D. from three experimental replicas.

4-HNE is a compound produced by the peroxidation of lipids within cells and is considered a second messenger of OS [35]. For the evaluation of 4-HNE expression (Figure 5B and Figure 6B), an immunohistochemical assay was performed in the middle and distal portions of the colon since reports [50,51,52] indicated that this is where the highest number of tumors occurs. To assess the effect of adding CC on the mice’s diet, we analyzed the percentage of expression of the marker (% 4-HNE expression) on Weeks 1, 7, and 14. Results of the % 4-HNE expression (Figure 5A and Figure 6A) indicated it was mainly found towards the lumen of the intestine and in the layer known as muscularis. The % 4-HNE expression in the three groups treated with carcinogens, compared to the NC, was 4, 9, and 8 times higher in the distal part and 2, 6, and 4 times higher in the middle part of the colon, on Weeks 1, 7, and 14, respectively. Additionally, in the distal portion of the colon, this marker was higher during Weeks 7 and 14 of the test. On the other hand, compared to the PC, the % 4-HNE expression in the middle and distal part of the colon significantly decreased during the three evaluated times in the AOM/DSS + 10% CC and AOM/DSS + 20% CC groups. Regarding the expression of 4-HNE in the groups not treated with carcinogens and who maintained diets added with 10 and 20% of CC, these showed concentrations similar to the NC in each analyzed time. Therefore, we proposed that diets with 20% CC inhibit the expression of oxidation markers, which could indicate that the compounds present in the CC exert a chemopreventive action.

## 4. Discussion

There were no significant changes in the major components of the CC with respect to the RC. In the case of proteins, the increase was similar to the results of Avola et al. [3], who reported a 6% increase attributed to a loss of soluble solids during cooking, which caused an increase in the protein concentration [38]. However, it has been observed that the behavior in the protein concentration, after the cooking process, depends on the type of legume. For example, it has been reported that in some varieties of *Phaseolus vulgaris*, the protein concentration decreased, while in *Lens culinaris*, it significantly increased [53]. Another report indicated that in these macro compounds, the main changes were not in the concentrations but in the digestibility and bioavailability of nutrients [54]. Villa et al. [55] reported that after heat treatments, allergenicity is decreased, and proteins were denatured, leaving the peptide bonds and the digestive enzyme recognition sites more exposed, making it easier to break the bonds. Additionally, there was also a certain degree of hydrolysis favored by heat, which could increase the antioxidant activity of proteins due to the increased exposure of R groups and the release of peptides with antioxidant activity [55,56]. A similar effect was seen in carbohydrates; Chinedum et al. [57] reported that in cooked beans, there was no significant loss in quantity, although the most soluble ones such as α-galactosides decreased. Additionally, these authors evidenced a decrease in the glycemic index, as a consequence of chemical modifications in the molecules due to the effect of heat. The authors also proposed that cooking the seeds softened the cell wall and other components of the cells, such as vacuoles and apoplast, releasing reserve compounds and causing them to interact; therefore, food was nutritionally and functionally enriched. For example, starches were more digestible after cooking [57]. Other reports [58,59,60] indicated that the bioavailability of folate in peas and beans increased after certain processes, including cooking and boiling. Folate is involved in tissue growth and cellular processes and its consumption improves the digestibility and absorption of iron.

As to non-nutritional compounds, it is necessary to reduce their concentration since they reduce the digestibility of food and give it an astringent flavor. While cooking for 25 min in a pressure cooker, a significant decrease in these compounds was observed due to their thermolability or solubility in water. It has been reported that phenolic compounds decreased up to 50% due to the effect of cooking, as a consequence of the high temperature and the destruction of the structural integrity of the plant tissue, in addition to the fact that the glycosylated molecules (for example, rutin, 3-glycosylated delphinidin, and quercetin) are hydrolyzed, favoring the generation of simple phenolic compounds [8,61]. A reduction of 14–17% in the concentration of saponins in chickpea seeds has also been reported after a soaking and cooking process. This value is lower than the one shown here and may be related to its amphiphilic nature, variety, growing conditions, and seed age [62,63]. In the case of phytates, their loss has been attributed to the formation of insoluble complexes by phytates and calcium or magnesium during thermic treatments [64]. Further, chemical hydrolysis of phytic acid to its less phosphorylated forms was observed in processes at 121 °C, suggesting instability of phytic acid at high temperatures [65].

Although it is necessary to decrease non-nutritional compounds’ concentrations, it is desirable to keep part of them, since their presence in lower concentrations in legumes confers their pharmacological capacity and reduces their toxicity. Among other relevant biological aspects, non-nutritional compounds possess antioxidant activity mainly due to phenolic compounds (condensed tannins, flavonoids, and anthocyanins) [35], saponins [49,66], phytates [67], protease inhibitors [68], peptides [69], as well as the non-digestible fraction, consisting mainly of carbohydrates [35].

The in vitro evaluation of antioxidant activity showed a significant decrease in each of the tested assays, which can be correlated with the partial loss of phenolic compounds (Table 1). However, CC still maintained between 62 and 68% antioxidant activity with respect to the raw seed (RC). Polyphenols have been reported to have the ability to trap free radicals and chelate metals [70], which is why their consumption is beneficial in chronic non-transmissible diseases, most of which are closely related to OS [71,72].

The antioxidant activity of chickpea has been correlated with its proteins, especially with some His-rich peptides encrypted within them [73]. These peptides have shown the ability to chelate metals (Cu^2+^ and Fe^2+^) and inhibit the oxidation of β-carotene in the presence of copper. Other peptides with molecular weights between 200 and 3000 Da, rich in Arg, Phe, Lys, Leu, Ala, and Asp, are capable of inhibiting the hydroxyl (OH^−^), superoxide (O_2_), and 2,2-diphenyl-1-picrylhydrazyl (DPPH) radicals, as well as preventing the oxidation of linoleic acid [44]. Additionally, chickpea protein hydrolysates have been reported to increase antioxidant enzyme activity (catalase, glutathione reductase, and glutathione peroxidase) in CaCo-2 and HT-29 cell lines [74]. Other studies have reported that saponins and phenolic compounds (shikimic and chlorogenic acids, rutin, daidzein, genistein, and biochanin A) from chickpea have shown antioxidant activity [75,76].

Mecha et al. [77] and Ombra et al. [78] reported that after a soaking and cooking treatment of different varieties of common beans, there was a reduction of up to 50% in phenolic compounds. However, they still had ORAC, attributed to the fact that after cooking, there was a higher proportion of flavanols and flavonoids in the seed, due to the softening caused by hydration.

Ombra et al. [78] reported that despite a loss of total polyphenols, there still was antioxidant activity after cooking since the quality/quantity ratio of phenolic compounds increased. Xu and Chang [79] reported that after cooking the chickpea seed, the antioxidant activity (measured by ORAC and DPPH) decreased. The authors attributed the decrease to the solubility and thermosensitivity of some phenolic compounds. They also related the drop in this activity to the increase in cooking time. However, despite the decrease, the cooked seed still presented antioxidant activity. The authors mentioned that the remaining antioxidant activity might be linked to the formation of aglycones, which are the product of the degradation of flavonoid glycosides. This behavior was attributed to chemical rearrangements caused by the release of hydrogen atoms that can be used to stabilize oxidation–reduction reactions. The reactions involve oxygen atoms or electron transfer mechanisms from the remaining phenolic compounds. This phenomenon has also been reported in faba beans, whose mechanism is the elimination of free radicals and polymerization of tannins and proanthocyanidins [80,81].

Phytic acid, like phenolic compounds, has also been reported to have antioxidant activity. It has been demonstrated to have a chelating effect on pro-oxidant minerals, such as iron, and anticarcinogenic potential [82]. Kapral et al. [83] reported that phytic acid could regulate proliferation and apoptosis markers in colon cancer cells by suppressing the expression and activity of key components such as AKT/mTOR (serine/threonine-protein kinase and mammalian target of rapamycin), AKT1 kinase, and p70S6K1 (ribosomal protein S6 kinase β-1). Other authors have attributed the antioxidant activity to the presence of peptides rich in hydrophobic amino acids and acidic and basic amino acid residues of legumin, a protein found in high concentration in chickpea. These peptides appear when gastrointestinal enzymes release them [84].

Antioxidant activity has been associated to anticancer therapies because OS is closely linked to the development of cancer. OS can activate a variety of transcription factors involved in the development of malignant tumors. In fact, the onset and progression of cancer have been linked to OS by increasing DNA mutations or damage, genome instability, and cell proliferation [85]. Furthermore, the tumor microenvironment is also highly oxidizing; therefore, antioxidants are important in anticarcinogenic therapies [86]. OS increases the production of prostaglandin and interrupts glutathione peroxidase production, a key enzyme in the endogenous antioxidant system [87]. Therefore, the consumption of antioxidant foods such as chickpea is important.

For the in vivo antioxidant activity evaluation of CC, a colon cancer model with AOM/DSS that causes oxidative damage to DNA was used. The evaluation was divided into three periods (1, 7, and 14 weeks after induction) to observe the action of CC during the development of carcinogenesis. AOM was used as a cancer initiator since it induces O^6^-methylguanine adducts into DNA during replication, leading to G → A transitions [88]. The AOM applied together with an agent that produces inflammation in the colon (DSS) favored the development of neoplastic lesions due to the damage caused in the colon’s epithelial barrier; besides, an overexpression of oxidative biomarkers has been observed in the PC of AOM/DSS for 8–12 weeks [89,90,91]. The results showed that the AOM/DSS group had a higher concentration of products derived from protein oxidation (CO) and nitrites that indicated oxidative damage, which peaked on Week 7. By Week 14, these compounds showed decreased production.

Regarding the AOM/DSS + 10% CC and AOM/DSS + 20% CC groups, NO, MDA, and CO concentrations showed a significant decrease. The NO concentration in the colon of the AOM/DSS + 10% CC and AOM/DSS + 20% CC groups was significantly lower compared to the PC. NO is a molecule commonly produced in the body for the regulation of various biological processes. Therefore, excessive production generates a relaxation of the arteries and a decrease in blood pressure; this is usually critical in ill people. Furthermore, this excessive production will have a direct action on proteins or DNA with other radicals, which will generate oxidative chain processes that can be minimized with antioxidant systems [92]. In the colon, motility regulation depends on NO-mediated enteric inhibitory neurotransmission, purine neurotransmitters, and neuropeptides [93]. NO is produced in dependent and independent ways. In the dependent form, three isoenzymes are present, the endothelial NO synthase [eNOS], neural NOS [nNOS], and iNOS. The latter is related to pro-inflammatory cytokines such as tumor necrosis factor α [TNF α], interleukin 1 (IL-1), and interferon γ (IFN-γ), so its overexpression must be regulated [94]. Increased NO synthesis by iNOS is a process that occurs during intestinal inflammation [95], which occurred in the experimental animals due to the presence of DSS.

Milán-Noris et al. [96] studied the anti-inflammatory effect of CC protein concentrates and methanolic (phenolic) extracts by accumulating nitrite as an indicator of NO synthesis in the macrophage cell line RAW264.7 by the Griess reaction. They found that CC methanolic extracts had greater anti-inflammatory activity than protein concentrates, inhibiting more effectively NO production. Additionally, Masroor et al. [97] analyzed the anti-inflammatory effect of methanolic extracts of *Cicer arietinum* L. in doses of 200 and 400 mg/kg in rats, concluding that the extracts had significant anti-inflammatory potential. Therefore, the effect shown in the present work may be due to a synergistic effect between the compounds present in the CC. On the other hand, in the groups that only received an added diet with 10 and 20% CC, there was no significant difference (*p* < 0.05) in NO concentrations compared to that of the negative control. This demonstrated that CC did not cause overexpression of NO, showing any diarrhea or constipation in the mice.

The consumption of various plant foods is unlikely to result in the production of antioxidant compounds in toxic amounts to the body. It has been proposed that the high antioxidant and anticancer activity of phytochemicals is due to the different types and concentrations in which they are found in plant foods. As a result, synergistic or additive effects are generated in their bioactivity [98]. However, combining two or more phytochemicals does not always improve the desired effect since antagonistic effects can be generated. By combining two or more phytochemicals, an effect is obtained that can be equal (additive effect), greater (synergistic effect), or less (antagonistic effect) than the sum of the individual effects of each compound in the mixture [29]. The synergistic anticancer effect has been observed with different bioactive compounds derived from natural plants, such as arctigenin and quercetin, and also in apple extracts enriched with phytochemicals [99].

The intake of phytochemicals from legumes such as beans, chickpeas, soybeans, and lentils, can confer beneficial effects on health by protecting against cardiovascular diseases (hypertension) and inflammatory processes. Its effect will be determined by the synergism or antagonism produced by the mixtures of phytochemicals present in legumes [100].

Regarding CO, these are the product of the oxidation of proteins. This process alters the conformation, activity, and function of proteins, making them highly resistant to proteolysis. Therefore, protein oxidation affects the functional integrity of cells during diseases [101]. In this study, the AOM/DSS + 10% CC and AOM/DSS + 20% CC groups were found to have significantly (*p* > 0.05) less CO concentration compared to PC on Weeks 7 and 14 of the experiment. While in Week 1, only AOM/DSS + 20% CC presented a lower concentration. It is essential to indicate that the increase in CO is related to inflammatory processes, since chronic inflammation increases the generation of ROS [102]. Luna-Vital et al. [103] evaluated the antineoplastic potential of an extract of peptides from the non-digestible fraction of common bean cv. Azufrado-Higuera tree and its most abundant peptide (GLSTK) in a colon cancer model with AOM/DSS in BALB/c mice. They observed that common bean peptides decreased inflammation and neoplasm formation in the colon of mice with AOM/DSS. Likewise, a study that used extracts of isoflavones (11 extracts) from chickpea sprouts enriched with selenium (4 days/24 °C) showed its capacity to absorb oxygen radicals, which can decrease CO generation; therefore, the authors suggested that they could be used in the treatment of colon cancer [104].

In the same way, the generation of OS will produce peroxidation of polyunsaturated fatty acids giving as final products MDA and 4-HNE. At the same time, it will worsen free radical chain reactions, alter the integrity of the intestinal mucosa barrier, and activate inflammatory mediators [105,106]. This was evident in the quantification of MDA concentration and % 4-HNE expression, as both are lipoperoxidation markers. The results obtained showed that the AOM/DSS + 10% CC and AOM/DSS + 20% CC groups had a significant decrease in MDA concentration versus the PC. Sánchez-Chino et al. [13] reported that the consumption of 2 and 10% CC diets reduced MDA and CO concentrations in groups administered AOM/DSS after 20 weeks of experimentation.

On the other hand, antioxidants and anti-inflammatory agents present in ulcerative colitis have been shown to decrease MDA concentrations and increase superoxide dismutase levels [105]. Rehman et al. [107] found that tannic acid (TA) applied at doses of 50 and 100 mg/kg body weight in a 1,2-dimethylhydrazine model in the colon of Wistar rats significantly decreased the MDA concentration. The authors attributed this to the fact that TA can exert antioxidant, anti-inflammatory, and antiproliferative activity. Larrosa et al. [108] used a mixture of 18 polyphenols in a rat DSS colitis model and observed that hydrocaffeic acid generated the most significant decrease in the colon MDA concentration. They related this to the direct antioxidant activity or the ability of this compound to increase the expression of eNOS. Guan et al. [109] reported a decrease in 4-HNE concentrations after consumption of δ-tocopherol and γ-tocopherol (0.2%) in a model of colon carcinogenesis in rats. They reported that the most effective treatment was δ-tocopherol since it presented a reduction in marker expression of 56.9%, followed by 39.9% with γ-tocopherol.

For their part, Monk et al. [45] evaluated a diet added with 20% CC flour to observe the behavior of the microbiota in the colon. They found that adding CC to the regular diet for three weeks improved the intestinal barrier by modulating the function of the colonic microenvironment due to the content of phenolic compounds and fermentable carbohydrates. Furthermore, the severity of the inflammatory response was reduced, since the activation of NF-κB in the colon tissue and the production of pro-inflammatory cytokines (TNF-α and interleukin IL-18) decreased, while the expression of anti-inflammatory molecules increased (IL-10, IL-22, and IL-27) [110].

## 5. Conclusions

The consumption of legumes and specifically chickpea represents an alternative for the prevention of chronic degenerative diseases since it is a source of compounds with antioxidant activity, even after being minimally processed (cooking). The substitution of 10 or 20% of CC in the diet decreased the concentration of NO, CO, MDA, and 4-HNE in the groups induced with AOM/DSS compared to the PC. The best effect was obtained with the diet added with 20% CC. The results obtained confirmed the antioxidant and anti-inflammatory activity of chickpea. This activity may be due to one or more compounds in the food or to synergism, for example, phenolic compounds, saponins, bioactive peptides, soluble, and insoluble fiber, among others. With the results obtained, we proved that chickpea is a good alternative for chemoprevention. Additionally, we demonstrated the importance of studying a whole seed since its consumption, use, accessibility, and impact on the nutritional and health status are promoted.

It is important to mention that in this study, there were certain limitations since an in vivo model was used, to which whole chickpea seed was administered, whose composition is complex. For this reason, it is not possible to attribute the observed effect to a specific compound. Therefore, it is advisable to carry out studies of isolated compounds and mixtures of two or more of them, to observe the effect obtained and later correlate it with the results obtained in studies like this one. However, it must not be forgotten that whole foods confer greater benefits than their isolated compounds. In countries whose diet is mainly based on plant foods, it is essential to understand the bioavailability of micronutrients and phytochemicals present in them. Therefore, it is important to study food synergies and the effect that processes, such as cooking, have on bioavailability.

We have shown that the consumption of chickpea has antioxidant activity on the oxidation of lipids and proteins. Therefore, within the perspectives of this study, it would be interesting to know what happens upstream and downstream, for example, when studying the effect of chickpea consumption on pro- and anti-inflammatory interleukins and on the expression of factors such as TNF-α and VEGF, as well as to evaluate its effect on other organs, the microbiota, and microenvironment.

## Figures and Tables

**Table 1:** Nutritional and non-nutritional chemical analysis of raw (RC) and cooked chickpea flours (CC).

	RC	CC
Moisture *	7.8 ± 0.30 ^a^	2.9 ± 0.09 ^b^
Ash *	2.83 ± 0.06 ^a^	1.22 ± 0.28 ^b^
Lipid *	7.11 ± 0.28 ^b^	9.76 ± 0.10 ^a^
Protein (NX5.8) *	25.17 ± 1.65 ^a^	27.32 ± 1.78 ^a^
Fiber *	1.71 ± 0.4 ^a^	1.41 ± 0.5 ^a^
Carbohydrates *	63.07 ± 1.65 ^a^	60.28 ± 1.7 ^a^
Saponins ^1^	1.78 ± 0.00 ^a^	1.25 ± 0.01 ^b^
Phytates ^2^	249.33 ± 10.1 ^a^	202.33 ± 6.5 ^b^
Trypsin Inhibitors ^3^	12.11 ± 0.02 ^a^	1.88 ± 0.03 ^b^

* g/100 g of seed on a dry basis, ^1^ mg diosgenin eq./g, ^2^ mg Phytic ac eq./100 g, ^3^ UIT/mg. Different letters per line indicate significant difference (*p* < 0.05) between the compounds by Tukey’s test. Values are presented as the mean ± S.D. from three experimental replicas.

**Table 2:** Antioxidant properties of raw (RC) and cooked (CC) chickpea.

	TPC ^1^	Antioxidant Activity		
		ORAC ^2^	OH^−^ Radical ^3^	Superoxide Radical ^4^
RC	60.09 ± 4.17 ^a^	52.73 ± 0.96 ^a^	56.36 ± 1.54 ^a^	57.05 ± 1.92 ^a^
CC	45.44 ± 2.32 ^b^	27.32 ± 1.22 ^b^	38.42 ± 2.01 ^b^	35.03 ± 1.76 ^b^

^1^ mg gallic acid eq./100 g, ^2^ mg Trolox eq./g dry base, ^3^ % OH-scavenging activity, ^4^ % O^2^-anion-scavenging activity. Different letters per column indicate significant difference (*p* < 0.05) between the compounds by Tukey’s test. Values are presented as the mean ± S.D. from five experimental replicas.
